# Default Mode Network Connectivity Predicts Emotion Recognition and Social Integration After Traumatic Brain Injury

**DOI:** 10.3389/fneur.2019.00825

**Published:** 2019-08-09

**Authors:** Katie Lancaster, Umesh M. Venkatesan, Jean Lengenfelder, Helen M. Genova

**Affiliations:** ^1^Kessler Foundation, West Orange, NJ, United States; ^2^Department of Physical Medicine and Rehabilitation, Rutgers New Jersey Medical School, Newark, NJ, United States; ^3^Moss Rehabilitation Research Institute, Elkins Park, PA, United States

**Keywords:** traumatic brain injury, emotion recognition, resting state functional connectivity, default mode network, community integration, TBI, DMN

## Abstract

Moderate-severe traumatic brain injury (TBI) may result in difficulty with emotion recognition, which has negative implications for social functioning. As aspects of social cognition have been linked to resting-state functional connectivity (RSFC) in the default mode network (DMN), we sought to determine whether DMN connectivity strength predicts emotion recognition and level of social integration in TBI. To this end, we examined emotion recognition ability of 21 individuals with TBI and 27 healthy controls in relation to RSFC between DMN regions. Across all participants, decreased emotion recognition ability was related to increased connectivity between dorsomedial prefrontal cortex (dmPFC) and temporal regions (temporal pole and parahippocampal gyrus). Furthermore, within the TBI group, connectivity between dmPFC and parahippocampal gyrus predicted level of social integration on the Community Integration Questionnaire, an important index of post-injury social functioning in TBI. This finding was not explained by emotion recognition ability, indicating that DMN connectivity predicts social functioning independent of emotion recognition. These results advance our understanding of the neural underpinnings of emotional and social processes in both healthy and injured brains, and suggest that RSFC may be an important marker of social outcomes in individuals with TBI.

## Introduction

Our ability to perceive and understand the emotions of others is crucial to successfully navigating social interactions and living in a social milieu. Because emotion recognition is a core social cognitive process, those with deficits in this ability—for instance, due to psychiatric condition or disease status—tend to have poorer outcomes in many domains of social functioning, such as successfully communicating with others (1), maintaining occupation (2), and participating in the community (3). Similarly, there exists a spectrum of emotion recognition ability within the healthy neurotypical population whereby greater recognition ability is associated with social competence and maintaining peer relationships (4, 5).

Deficits in emotion recognition are a pervasive yet under acknowledged aspect of traumatic brain injury (TBI). It is estimated that up to 39% of individuals with moderate-severe TBI suffer from significant emotion recognition deficits, with the degree of impairment approximating a standard deviation difference from the performance of healthy individuals (6). In TBI, these emotion recognition impairments predict a number of social, cognitive, and behavioral issues such as deficits in self-awareness, behavioral inhibition and emotion regulation (7, 8), social communication (9), and social competence (7).

As the nature of injury in individuals with TBI is diffuse and heterogeneous (10), the specific neurobiological substrates of social cognitive deficits in TBI are still being identified. While some have examined these deficits using task-based fMRI experiments (11), and structural studies (12–14), there is growing interest in using the brain's intrinsic functional connectivity to explain deficits in TBI (15, 16). For instance, resting state functional connectivity (RSFC) can be used to predict cognitive and behavioral outcomes for individuals with TBI who do not have detectable anomalies in brain structure (17).

Of particular interest is the default mode network (DMN), a functional network that captured scientific interest when it was found to be robustly activated during periods of rest (18, 19). Aberrant functional connectivity within the DMN has been demonstrated in TBI, predicting impairments in cognition (20–22), and functional outcomes like depression and fatigue (22). Furthermore, the DMN is particularly relevant to social cognition as the regions comprising the DMN are also engaged during social and emotional processes (23, 24). It has been argued that DMN RSFC may represent a neuromarker of individual differences in social abilities, predicting mentalizing ability in neurotypicals (25), predicting autistic traits in neurotypicals and people with autism (26), and predicting social network size in macaques (23) and humans (27). While DMN RSFC is an important and flexible tool for investigating social cognition and behavior, it is currently unclear how it relates specifically to the component process of emotion recognition ability. Although the regions comprising the DMN have been linked to emotion recognition in task-based paradigms (28), there is little work examining individual differences in emotion recognition using DMN RSFC. It is unknown how RSFC patterns within the DMN are related to emotion recognition ability in healthy neurotypicals, how these relationships may differ in TBI, and whether they are predictive of socially-relevant functional outcomes.

To address these gaps in the literature, the current work investigated RSFC within the DMN (hereafter referred to as “DMN connectivity”) in relation to emotion recognition in healthy individuals and those with moderate-severe TBI. We hypothesized that DMN connectivity would be associated with individual differences in emotion recognition ability, and further examined whether these relationships were altered in the context of TBI. We also sought to test the hypothesis that emotion recognition deficits contribute to social functioning problems after TBI by examining the extent to which emotion recognition ability predict a real-world measure of post-injury community integration. Furthermore, as prior work has suggested that RSFC may be independently related to social functioning (29, 30), we will test whether DMN connectivity is a stronger predictor of community integration than emotion recognition ability—this finding would suggest that DMN connectivity could be used within rehabilitation research as a predictive tool or as a treatment target.

## Methods

### Participants

A total of 53 people participated in the current research (25 TBI and 28 healthy controls [HC]). Participants with TBI were identified through our participant database, which comprises individuals recruited originally from local hospitals and the general community. Eligible participants sustained a single, closed-head moderate or severe TBI. The severity of the TBI was determined using the Mayo Classification System criteria (31), which for the current study were any of the following: (1) loss of consciousness for 30 min or more, (2) post-traumatic anterograde amnesia for 24 h or more, (3) lowest Glasgow Coma Score in the first 24 h ≤ 12, or (4) evidence of significant neurological injury on CT/MRI (e.g., subdural hematoma, cerebral contusion, subarachnoid hemorrhage). Injury severity was confirmed from medical records when possible; in the absence of medical records, severity was determined family member attestations of the length of loss of consciousness/coma. Participants were deemed eligible for the study if they were at least 12 months post injury. Injury characteristics for participants with TBI are presented in Table 1. HC participants were recruited from the general community and had no history of head trauma or neurological disorder. Four participants (1 HC and 3 TBI) were excluded due to excessive head motion, and one TBI participant was excluded due to an outlying low score on the emotion recognition task (more than three standard deviations from the mean), leaving a final sample of 21 TBI and 27 HC. Participant groups did not significantly differ on mean age or education, or sex distribution as seen in Table 2. Participants completed behavioral measures (neuropsychological testing, social cognitive tasks, and self-report questionnaires) in an initial testing session and were scanned ~1 week later at an adjacent imaging facility [*M* = 8.44 (10.61) days]. All subjects gave written informed consent in accordance with the Declaration of Helsinki. The protocol was approved by the Kessler Foundation Institutional Review Board.

**Table 1:** Injury characteristics for TBI participants.

**Nature of injury**	**GCS score**	**PTA**	**LOC**	**Neuroradiological findings**
Fall	8			Epidural hematoma
MVA	13			Cerebral contusion
Unknown			>30 min	
Fall	5			Subarachnoid hemorrhage, cerebral contusion
MVA	6			Hemorrhagic contusion, subarachnoid hemorrhage
Fall	9			Subdural hematoma, intracerebral hemorrhage, subarachnoid hemorrhage
MVA			6 weeks	
MVA	4			
Assault			>30 min	
Fall	14	~36 h		
MVA	13			Epidural hematoma; subdural hematoma, subarachnoid hemorrhage
MVA	3			
MVA				Diffuse axonal injury
motorcycle accident			>30 min	
MVA	11			Cerebral contusion
Fall				Epidural hematoma
Fall	15			Subarachnoid hemorrhage
Fall	15			Subarachnoid hemorrhage, multiple contusions
MVA			35 days	
Struck by vehicle				Intracerebral hemorrhage, subdural hemorrhage
MVA	3			Subdural hemorrhage, intracerebral hemorrhage

*GCS, Glasgow Coma Scale; PTA, post-traumatic amnesia; LOC, loss of consciousness; MVA, motor vehicle accident*.

**Table 2:** Demographic and performance information for study participants.

	**TBI**	**HC**	***t***	***p***
	**Mean (*SD*)**	**Mean (*SD*)**		
**Demographics**				
Age	41.71 (15.22)	38.00 (13.66)	0.89	0.379
Education	14.64 (1.92)	15.48 (1.93)	−1.50	0.141
Months since injury	112.47 (97.95)	–	–	–
			*x^2^*	*p*
Gender	3F/18M	9F/18M	2.29	0.185
**Performance**				
TASIT performance	22.52 (2.60)	24.78 (1.72)	−3.61	0.001
Cognitive composite score	0.30 (0.76)	−0.38 (0.78)	−3.00	0.004

### Assessment of Emotion Recognition

We employed a measure that has previously been validated for use in the TBI population, The Awareness of Social Inference Test [TASIT; (32)], which includes multiple subtests that tap into different aspects of social cognitive ability. In the current study, we examined the TASIT Emotion Evaluation Task, which assesses emotion recognition ability via a sequence of short (15–60 s) videotaped vignettes featuring interactions among trained actors. Participants were instructed to view each vignette and identify which emotion was being conveyed by the actor from a choice of seven basic emotions (neutral, surprised, anxious, sad, angry, revolted, and happy). Four instances of each emotion were presented in vignettes in a pseudorandomized sequence; this presentation order was consistent across all participants. A sum of correct responses for all trials was computed with a maximum attainable score of 28.

### Neuropsychological Assessment

Participants completed a battery of neuropsychological tests sensitive to the primary neurocognitive deficits seen in TBI, including processing speed, attention, and executive functioning. These cognitive domains also have been shown to influence aspects of social cognition across various clinical disorders [e.g., (33–35)]. Therefore, we examined the potential influence of neuropsychological performance on emotion recognition analyses with a cognitive composite score, which was obtained by averaging z-scored performances on tests of processing speed, working memory, and executive functioning. Constituent tests included Block Design from the Wechsler Abbreviated Scale of Intelligence-II (36), Trail Making- Number-Letter Switching Condition and Color-Word Interference- Inhibition Condition from the Delis-Kaplan Executive Function System (37), and the Symbol Digit Modalities Test (38). This cognitive composite score was entered as a covariate in functional connectivity analyses.

### Measurement of Community Integration

The Community Integration Questionnaire [CIQ; (39)] was designed for use with individuals with TBI to assess social integration, a fundamental component of recovery and rehabilitation that contributes importantly to positive post-injury outcome, including mental and physical health (40) and quality of life (41). This self-report questionnaire comprises three subscales, which index integration in home activities, productivity (employment or volunteer activities), and social activities. A widely used measure, the CIQ has demonstrated good validity and reliability within the TBI population (39, 42).

### Image Acquisition

Imaging data were acquired using a Siemens Magneton 3T Skyra scanner (Siemens Corporation, Erlangen, Germany). Echo-planar imaging (EPI) was used to image the resting state, during which participants were instructed to lay still with eyes closed. EPI data were acquired over the course of 6 min, with 32 images of 3 mm thickness aligned AC-PC (180 volumes, TR = 2,000 ms, TE = 30 ms, flip angle = 70°, voxel size = 2.3 × 2.3 × 3 mm). Additionally, a high-resolution anatomical image was acquired for ~5 min, with 176 slices of 1 mm thickness (TR = 2,100 ms, TE = 3.43 ms, flip angle = 9°, voxel size = 1 mm isotropic).

### fMRI Pre-processing

Imaging data were pre-processed using Statistical Parametric Mapping software, SPM8 (Wellcome Department of Cognitive Neurology, London, UK). Functional images were pre-processed using a standard pipeline, including slice-timing correction for interleaved slice acquisition, realignment of the image series to the first functional image, coregistration of functional, and structural images, tissue segmentation, and normalization of images to standard Montreal Neurological Institute (MNI) space using 12-parameter affine transformations and non-linear registration. Images were smoothed using a 6 mm FWHM Gaussian kernel to improve the ratio of signal-to-noise. Motion correction was then applied (see below), and noise signals were estimated and removed using linear detrending, a bandpass filter of 0.01–0.12 Hz, and the aCompCor procedure (43) as implemented in the CONN toolbox (44). This method removes effects of white matter (WM) and cerebrospinal fluid (CSF) on the BOLD signal using the participant-specific WM and CSF masks, while avoiding the augmentation of negative correlations between voxels associated with global mean signal regression (45).

### Default Mode Network (DMN) Connectivity

Functional connectivity analyses were performed with the CONN toolbox (44) using DMN regions of interest (ROIs) defined *a priori* from Power et al.'s cortical atlas (46). The DMN in this atlas comprises 58 ROIs in medial pre-frontal, posterior cingulate/precuneus, and bilateral temporal and temporoparietal regions. ROIs were defined as non-overlapping spheres of 10 mm diameter. In first-level analyses, BOLD timeseries were averaged across all voxels of each ROI and correlated with the remaining ROIs, such that for each participant, we obtained a DMN ROI-ROI correlation matrix. First-level correlations were then Fisher-transformed and subjected to second-level tests of ROI-to-ROI connectivity within the DMN, including (1) group differences (HC > TBI and TBI > HC) in connectivity and (2) correlation between emotion recognition ability and DMN connectivity across groups. All connectivity analyses employed FDR-correction (α = 0.05) to control for multiple comparisons. Additionally, we extracted first-level connectivity values for connections showing significant relationships with emotion recognition at the group level. Using SPSS, we entered these values into regression analyses predicting community integration in individuals with TBI [analyses were constrained to individuals with TBI as a reduction in community integration is a common sequela of TBI; (47)].

### Motion Artifacts

To correct for head movement, we used the ArtRepair toolbox (48), which addresses both multivolume and smaller motion perturbations. Following realignment, large amplitude motion correction was applied using trigonometric form adjustment. Rapid scan-to-scan motion was adjusted following normalization and smoothing. Volumes with more than 1 mm scan-to-scan movement (translation and rotation) were treated as artifacts and replaced with interpolated signal from adjacent, unaffected volumes. Participants with more than 20% artifactual volumes (four participants) were excluded from further analyses. In the remaining sample, TBI and HC groups did not differ on the number of artifactual volumes, *M*_*TBI*_ = *5.86 (9.33), M*_*HC*_ = *2.63 (6.70), t*_(46)_ = *1.42, p* = *0.16*.

## Results

### Group Differences in Emotion Recognition Ability, Cognition, and Connectivity

Compared to HCs, the TBI group demonstrated significantly reduced emotion recognition ability as measured by the TASIT, *t*_(46)_ = *3.61, p* = *0.001*, and cognitive performance, *t*_(46)_ = *3.00, p* = *0.004*. However, there were no significant group differences in DMN connectivity metrics examined at the second level (*p-FDR* > 0.05).

### Emotion Recognition Ability and DMN Connectivity

**Figure 1:**
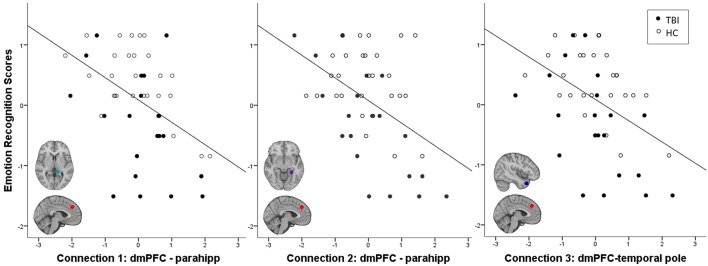
Negative relationship between emotion recognition ability and frontal-temporal connectivity strength. Z-normalized TASIT scores are plotted again first level fisher-transformed correlation coefficients denoting the strength of functional connectivity between regions described in Table 3. Parahipp = parahippocampal gyrus.

Across all participants, emotion recognition was inversely associated with connectivity between an ROI in dmPFC and three temporal lobe ROIs: two in left parahippocampal gyrus (parahipp) and one in right temporal pole (Table 3). Relationships between connection strength and emotion recognition scores are illustrated in Figure 1. There were no significant interactions between emotion recognition performance and group membership on DMN connectivity (all *p-FDR* values > 0.05).

**Table 3:** ROI-to-ROI connectivity associated with emotion recognition.

**Connection**	**ROI 1**	**ROI 2**	***t***	***p-FDR***
1	**dmPFC;** xyz = [−2, 38, 36]	**parahippocampal gyrus;** xyz = [−13, −40, 1]	−3.53	0.027
2		**parahippocampal gyrus/fusiform gyrus;** xyz = [−26, −40, −8]	−3.56	0.027
3		**temporal pole;** xyz = [46, 16,−30]	−3.34	0.032

*ROI, region of interest, dmPFC, dorsomedial prefrontal cortex, xyz, coordinates of the centroid voxel of each ROI reported in Montreal Neurological Institute (MNI) stereotaxic space*.

### Influence of Potential Confounding Variables on the Emotion Recognition-Connectivity Relationships

Emotion recognition ability was significantly correlated with general cognitive performance, *r*_(46)_ = *0.44, p* = *0.002*, age, *r*_(46)_ = −*0.51, p* < *0.001*, months since injury, *r*_(19)_ = −*0.45, p* = *0.039*, and was marginally associated with gender such that female participants had slightly higher recognition ability than males, β = −*0.27, t*_(46)_ = −*1.96, p* = *0.061*. Level of education was not associated with emotion recognition ability, *r*_(46)_ = *0.19, p* = *0.188*. To ensure that demographic variables and group status did not unduly influence the relationship between emotion recognition and DMN connectivity metrics, we tested these covariates in linear regression analyses with each of the three connectivity metrics as dependent variables. We found that controlling for the influence of these covariates did not change the associations between emotion recognition and DMN connectivity. Results of these analyses are described further in Supplementary Table 1.

### Connectivity Metrics and Community Integration

Finally, we were interested in whether the DMN connectivity metrics which we identified in the previous analysis could independently explain social functioning in TBI, over and above the influence of emotion recognition. To this end, we examined the relationships between the three DMN connections and TBI participants' level of community integration, controlling for emotion recognition. Indeed, within the TBI group, the first functional connectivity metric from Table 3 (dmPFC-parahipp) was significantly inversely associated with total CIQ score, *r*_(18)_ = −*0.60, p* = *0.005* (the second and third connectivity metrics did not significantly correlate with CIQ, both *p*s > 0.18*)*. Further, consistent with our previously reported finding (49), emotion recognition ability in the TBI sample was related to better community integration (total CIQ score), *r*_(18)_ = *0.44, p* = *0.051*. Thus, in order to test the incremental predictive value of the first DMN connectivity metric on community integration, controlling for participants' emotion recognition ability, we conducted a hierarchical multiple regression (see Table 4). Potential confounds such as gender (β = −*0.26, t*_(18)_ = −*1.12, p* = *0.276*), education [*r*_(18)_ = *0.135, p* = *0.572*], cognitive ability [*r*_(18)_ = *0.06, p* = *0.798*], and months since injury [*r*_(18)_ = −*0.03, p* = *0.914*] were not related to total CIQ score. However, age was a significant predictor of community integration [*r*_(18)_ = −*0.45, p* = *0.045*] and is thus treated as a covariate in the analysis. In the first model, we entered emotion recognition ability (TASIT performance) as a predictor and participant age as a covariate; the model was marginally significant, *F*_(2, 17)_ = *3.09, p* = *0.07*. In the second model, in addition to TASIT and age, we added the first DMN connectivity metric (dmPFC-parahipp) as a predictor and found that the overall model was significant, *F*_(3, 16)_ = *4.32, p* = *0.021*, that DMN connectivity was a significant predictor of CIQ, β = −*0.47, t*_(16)_ = −*2.29, p* = *0.036*, and explained an additional 18% of the variance. Furthermore, we examined the subscales of the CIQ and determined that this effect was being driven primarily by the Social Integration subscale, which was strongly associated with dmPFC-parahipp connectivity, *r*_(18)_ = −*0.58, p* = *0.008* (neither Home Integration nor Productivity were significantly related to functional connectivity, *p*s > 0.65). Together these data suggest that participants' frontal-temporal DMN connectivity at rest is predictive of the social aspects of community integration in TBI, and is a stronger predictor than their emotion recognition ability.

**Table 4:** Hierarchical linear regression testing associations between emotion recognition performance, DMN connectivity, and community integration.

		**Model statistics**		**Model change**				**Predictors**		
**Models**	**Predictors**	***F***	***p***	***R^**2**^***	***R^**2**^* change**	***F* change**	***p***	**β**	***t***	***p***
1	3.09	0.072	0.27	0.27	3.09	0.072				
Age							−0.31	−1.28	0.216	
TASIT performance							0.29	1.19	0.249	
2	4.32	0.021	0.45	0.18	5.25	0.036				
Age							−0.20	−0.89	0.387	
TASIT performance							0.18	0.82	0.426	
dmPFC-parahipp connectivity							−0.47	−2.29	0.036	

*The dependent variable is total score on the CIQ*.

## Discussion

Impaired emotion recognition is prevalent in TBI and has deleterious social consequences, yet the neurobiological correlates of this impairment remain poorly understood. The current study examined DMN connectivity in relation to emotion recognition and social functioning in a sample of individuals with TBI and HCs. We found that while there were no significant group differences in DMN connectivity, there was a relationship between emotion recognition ability and frontal-temporal connectivity strength across groups. Moreover, frontal-temporal connectivity was predictive of social integration in the TBI group, even more robustly than their emotion recognition scores.

Across both groups, we found that greater frontal-temporal DMN connectivity (specifically dmPFC-parahipp and dmPFC-temporal pole) was associated with worse performance on an ecologically valid measure of emotion recognition, the Emotion Evaluation subtest of the TASIT (32). This result is consistent with a recent study in healthy individuals that found greater connectivity (specifically between the posterior DMN—inclusive of dmPFC—and regions including parahippocampal gyrus and temporal pole) was associated with worse performance on a test of emotion intelligence incorporating emotion recognition (50). The extension of these findings in the current study to include individuals with neurologic compromise suggests that the injury-related pathophysiology contributing to emotion recognition deficits in TBI may lie at one end of the physiological continuum that also characterizes individual differences in healthy controls.

Altered DMN connectivity in relation to emotion recognition is also consistent with a larger literature describing dysregulation within and between DMN subsystems in a variety of mental health disorders involving social cognitive deficits (29, 51–54). Importantly, cognitive neuroscience studies have identified DMN subsystems, anchored in part by distinct portions of the mPFC, that are involved in social processing (55). A dorsal mPFC subsystem—which shows strong connectivity with lateral cortex such as inferior frontal gyrus and temporoparietal junction—is involved in abstract social processing and mentalizing, whereas a ventral mPFC subsystem—tightly coupled with hippocampus and limbic regions—is involved in introspective thought driven by motivational and emotional states (56–58). These subsystems are believed to interact dynamically during successful social cognitive processing (57), and thus reductions in their interplay should be detrimental to social functioning. Moreover, the greater the positive (or weaker the negative) correlation between brain regions/networks has often been interpreted as reflecting a loss of network interplay [for a broader account of this functional “dedifferentiation” see, e.g., (59)]. Therefore, our findings may reflect emotion recognition failures associated with less differentiated activity of dorsal and ventral mPFC DMN subsystems, represented in our study by the dMPFC and parahippocampal cortex, respectively.

While a relationship between increased connectivity and reduced behavioral performance may at first seem counterintuitive, we highlight that such “hyperconnectivity” —particularly within the DMN—has been reported in several previous studies of moderate-severe TBI (60–65) as well as other neurologic disorders (66), although its functional significance has remained unclear. For example, it has been proposed that increased connectivity arises as an indirect response to structural disruption (61, 67), reflecting neural communication through alternative (and less efficient) pathways due to degraded direct connections [(68, 69); see (70), for review]. In this light, increased within-DMN connectivity may reflect a neural (but not necessarily behavioral) compensation for reduced structural integrity, arising from injury or from natural variation in white matter (14, 71, 72).

Lending further credence to the functional relevance of DMN RSFC to social processing, we noted that within-DMN connectivity was predictive of social integration of individuals with TBI. This complements recent schizophrenia research that demonstrates RSFC between DMN nodes is predictive of social functioning and competence (29, 30). Importantly, these relationships are not mediated by social or cognitive deficits, indicating that RSFC metrics may be *more* powerful predictors of social functioning outcomes than behavioral measures. These results suggest that DMN connectivity metrics may ultimately hold some promise as biomarkers relevant to clinical management and rehabilitation of TBI. Several studies have shown that functional connectivity has prognostic value in predicting recovery from brain injury (20, 73–76). Neuroimaging metrics can also be used to predict response to rehabilitative efforts: for instance, Arnemann et al. (77) found that functional network organization of individuals with acquired brain injury predicted their degree of improvement from a cognitive training intervention, implying that baseline neuroimaging could be used to identify individuals who are most appropriate for treatment. Furthermore, while still nascent in its clinical application—there is accumulating evidence that these neuroimaging metrics could themselves be the target of intervention, as demonstrated by the use of neurofeedback in EEG and real-time fMRI to rehabilitate brain injury (78, 79). The findings from the current study could thus have emergent clinical relevance in guiding treatment for TBI, particularly as it applies to social functioning and integration. Given the critical need for improving social functioning in TBI and the growing number of interventionist approaches which target social cognitive deficits (80, 81), results of the current study could inform this important subset of brain injury rehabilitation research: DMN connectivity could serve either as a predictor of treatment response to interventions, or as the treatment outcome itself.

The current study should be interpreted in the context of certain limitations. First, in contrast to many studies characterizing RSFC disruptions in TBI (82), we did not find significant connectivity differences between groups (as illustrated in Figure 1, the TBI group trended toward showing increased frontal-temporal connectivity, but this was not significant). It is unclear whether this is attributable to the low power due to modest sample size (21 TBI), or to the difficulty in surviving multiple corrections due to the large number of regions in the DMN atlas we used for this study. However, in studies like ours which examine both group differences and individual differences, the regions that differ between TBI and HC are often not the same regions that covary with individual difference variables (e.g., 16, 66). Thus, the lack of significant group differences does not necessarily affect the interpretation of our individual difference findings. Another limitation from this study concerns its scope. Analyses from the current study were constrained to a single, theoretically motivated resting state network—the DMN. However, as the brain regions facilitating emotion recognition are not entirely limited to those found within the DMN (28), it is likely that our results would be more complex had we also examined other networks. For instance, Rigon et al. examined RSFC within a network of regions identified meta-analytically and found a distributed network (including intra- and inter-hemispheric connections) of frontal and temporal regions associated with emotion recognition ability in participants with TBI (16). Thus, while our results are not an exhaustive RSFC characterization of emotion recognition ability in healthy individuals or those with TBI, they provide a concise and theoretically informed illustration of the RSFC substrates of emotion recognition ability, and further demonstrate that these substrates can be used to predict social functioning in TBI.

## Conclusion

We present the first evidence of RSFC correlates of emotion recognition within the DMN, and show that these metrics can be used to predict social functioning in individuals with moderate-severe TBI. These findings highlight the importance of examining intrinsic functional networks and their contributions to complex social processes and behavior.

## Data Availability

The datasets generated for this study are available on request to the corresponding author.

## Ethics Statement

All subjects gave written informed consent in accordance with the Declaration of Helsinki. The protocol was approved by the Kessler Foundation Institutional Review Board.

## Author Contributions

HG and JL obtained grant funding to perform the study. All authors contributed to the study design, manuscript revision, and approved the submitted version. Data analysis was performed by KL and UV, and supervised by HG. The article was drafted by KL, UV, and HG.

### Conflict of Interest Statement

The authors declare that the research was conducted in the absence of any commercial or financial relationships that could be construed as a potential conflict of interest.
