# Supplementary material for: Default Mode Network Connectivity Predicts Emotion Recognition and Social Integration After Traumatic Brain Injury
Source: Front Neurol. 2019 Aug 9;10:825. doi: 10.3389/fneur.2019.00825 (PMC6696510; doi:10.3389/fneur.2019.00825)
Supplement: Supplementary file 1 [file Table_1.pdf]

Supplementary Table 1. Emotion recognition predicts DMN connectivity strength after controlling for covariates. Three linear regression models tested the association between emotion recognition ability (TASIT performance) and each of the DMN ROI-to-ROI connections described in Table 3.

	Dependent variable								
Predictor variables	**Connection 1** *dmPFC-parahipp*			**Connection 2** *dmPFC-parahipp*			**Connection 3** *dmPFC-temporal pole*		
	β	*t*	*p*	*β*	*t*	*p*	*β*	*t*	*p*
TASIT performance	-.61	-3.48	.001	-.58	-3.17	.003	-.47	-2.63	.012
Group status (HC vs. TBI)	-.16	-.83	.411	.04	.22	.824	.39	1.96	.057
Cognitive composite score	.37	2.15	.037	.30	1.65	.106	.14	.77	.444
Age	.32	1.88	.068	.22	1.23	.225	.04	.26	.800
Months since injury	-.30	-1.59	.119	-.07	-.37	.712	.31	1.59	.119
Gender	-.21	-1.41	.165	-.11	-.74	.466	.08	.50	.617
